# Limited value of long-term biochemical follow-up in patients with adrenal incidentalomas-a retrospective cohort study

**DOI:** 10.1186/s12902-015-0001-x

**Published:** 2015-02-27

**Authors:** Hannah Yeomans, Jan Calissendorff, Cristina Volpe, Henrik Falhammar, Buster Mannheimer

**Affiliations:** Department of Clinical Science and Education at Södersjukhuset, Karolinska Institutet, SE 118 82 Stockholm, Sweden; Department of Molecular Medicine and Surgery, Karolinska Institutet, SE 171 76 Stockholm, Sweden; Department of Endocrinology, Metabolism and Diabetes, Karolinska University Hospital, D2:04, SE 171 76 Stockholm, Sweden

**Keywords:** Adrenal tumour, Hypersecreting tumours, Functional, Follow-up

## Abstract

**Background:**

The prevailing view that advocates long-term hormonal follow-up of adrenal incidentalomas is currently under debate. The purpose of the present study was to examine all adrenal incidentalomas presented during five years to a single centre. We hypothesized that 24-month biochemical follow-up in patients with an initial normal screening would fail to increase the sensitivity in finding hormone producing tumours.

**Methods:**

The present study is a retrospective register based cohort study of 194 patients referred to the Department of Endocrinology at Södersjukhuset between the years 2006–2010. Computerized medical records were used to find and extract information on patients with newly discovered adrenal incidentalomas. The sensitivity, specificity, positive predictive value and negative predictive value were calculated to evaluate the validity of an initial normal screening when used to identify individuals with hormone producing tumours.

**Results:**

Of the incidentalomas 94% consisted of benign, non-functioning tumours. Three patients were diagnosed with cortisol hypersecretion and one with pheochromocytoma. The sensitivity, specificity, positive predictive value and negative predictive value of an initial complete negative screening to predict a hormone producing tumour were 100%, 63%, 12% and 100%, respectively.

**Conclusion:**

Patients with an initially normal hormonal screening may not need further biochemical follow-up.

## Background

An adrenal incidentaloma is a tumour in the adrenal gland accidentally discovered when a patient undergoes radiological investigation for other reasons than suspected adrenal disease [1]. The mean prevalence is 2.3% and increases with age to 6.9% in patients above 70 years [2]. The prevalent occurrence together with the increased use of radiological investigation has resulted in the frequent incidental discovery of asymptomatic adrenal masses. Even though the majority of incidentalomas are benign and non-hyperfunctioning, careful radiological and hormonal investigations are needed in order to identify malignant and hormone producing tumours [3]. The management often includes biochemical hormonal surveillance maintained for several years which may be associated with significant emotional distress [4] and financial [5] costs in a group of patients where the vast majority turn out to be healthy. The prevailing view that advocates long-term hormonal follow-up [3,6,7] has therefore recently been challenged [8]. However, the evidence remains scarce. The present study examined all adrenal incidentalomas presented to a single centre between 2006 and 2010. We hypothesized that long-term biochemical follow-up in patients with an initial normal biochemical screening would fail to increase the sensitivity in finding hormone-producing tumours.

## Methods

### Consent statement

This was a retrospective database study that included data on 294 patients treated at Södersjukhuset during a period of 5 years. Hence, we did not interfere with the treatment of these individuals nor in any other way. This view was also supported by the Regional Ethics Committee in Stockholm, Karolinska Institute, which waived the need for written informed consent from the participants and approved the study as a whole.

### Setting and participants

The present study is a retrospective register based cohort study of patients referred to the Department of Endocrinology at Södersjukhuset between the 1^st^ of January 2006 and the 31^st^ of December 2010. Södersjukhuset is a hospital providing secondary, emergency and elective care to 600 000 inhabitants in Stockholm, Sweden. All patients with a new incidentally discovered adrenal mass was selected. Computerized medical records were used to find patients with any of the International Classification of Diagnoses (ICD) 10 codes D441 (tumour of unclear origin in adrenal gland), D350 (benign tumour in adrenal gland), E279 (adrenal disease unspecified) or C741 (malignant tumour in adrenal gland) were screened for possible inclusion. Patients with previously known adrenal tumour/s or who were investigated on suspicion of adrenal disease, and patients with previously known co-existing malignancy or signs of extra adrenal malignancy at baseline were excluded. Patients with a previous diagnosis of malignancy, but no evidence of present malignancy at the time the adrenal incidentaloma was detected were eligible for inclusion. The Swedish national guidelines during the studied period recommended initial radiological and hormonal evaluation and (if both normal) to be followed by a new biochemical evaluation 24 months later. The hormonal evaluation comprised of investigating cortisol and catecholamine secretion in all patients. In patients with hypertension or hypokalaemia measurement of aldosterone and renin secretion was added (see below). To improve the evaluation of the 24-month follow-up, patients who had not completed the investigation were approached and offered to do so. Fourteen patients subsequently underwent complementary testing. Follow-up information was collected until the 31^st^ of October 2013.

The study was approved by the Ethics Committees of the Karolinska Institutet, Stockholm, Sweden.

### Variables and datasources

All information in the present study, including the biochemical screening and radiological tumour characteristics (tumour size, attenuation and washout) from the initial visit as well as from follow-up visits, were collected from the computerized medical files. Existence of co-morbidity was only registered for the initial visit. The biochemical data was retrieved from the laboratory database and included: ^a/^ aldosterone, evaluated using the aldosterone/renin ratio (ARR), ^b/^ catecholamine secretion evaluated by measurement of plasma metanephrines or a 24-hour urine collection of catecholamines, and ^c/^ evaluation of cortisol secretion. The initial screening for cortisol hypersecretion was performed using the 1 mg overnight dexamethasone suppression test (DST) (n = 107) or a 24-hour urine collection of cortisol (n = 45). In patients with deviating results of this initial screening and/or with clinical signs of cortisol hypersecretion, one or several additional tests were assessed. A cortisol hypersecretion was defined as any two of the following tests being abnormal: ^a/^ DST, ^b/^ a 24-hour urine collection of cortisol, ^c/^morning plasma adrenocorticotropic hormone (ACTH) and, ^d/^ a circadian rhythm of serum cortisol in the absence of evidence of severe stress, alcoholism, depression, or drug-drug interactions. Cushing’s syndrome was defined as clinical signs typical of Cushing’s syndrome (moon face, buffalo hump, hypertension, osteoporosis, diabetes mellitus) together with two tests of cortisol secretion being abnormal. Subclinical Cushing was defined as two abnormal tests in an individual without clinical signs of Cushing’s syndrome.

### Laboratory methods and cut-off values

All analyses were performed at the Karolinska University Laboratory. Serum cortisol was measured by ElectroChemiLuminiscence Immunoassay (ECLIA) (Roche Diagnostics). DST was considered normal when morning cortisol fell below 50 nmol/L. The circadian rhythm was performed in hospitalised patients and considered normal when the midnight cortisol value was below 50 nmol/L. Urine cortisol was measured by high performance liquid chromatography (Waters Quattro Premier MS/MS-system with Waters Acquity UPLC). The normal range was 40 nmol/d – 170 nmol/24 h. Plasma renin was assessed with the Electrabox CISBIO IRMA kit. ACTH was measured by ElectroChemiLuminiscence Immunoassay (ECLIA) (Roche Diagnostics). Morning ACTH concentrations <2 pmol/L were considered subnormal. Plasma aldosterone was determined using Siemens Coat-A-Count RIA kit (Siemens Ltd). An ARR >100 was considered to indicate autonomous aldosterone production [9,10]. The 24-hour urine collections of catecholamines were measured by high performance liquid chromatography (HPLC). The normal range for urinary adrenalin and noradrenalin was <80 nmol/24 h and <400 nmol/24 h, respectively [11]. Fasting plasma metoxycatecholamines were assessed by reversed phase chromatography (LC-MS/MS Xevo QT with Acquity UPLC, from Waters Sweden AB). The normal ranges for p-metoxyadrenaline and p-metoxynoradrenaline were <0.3 nmol/L and <0.6 nmol/L, respectively.

### Radiological classification of adrenal lesions

Lesions were classified as benign if they were small (<3 cm) and had radiological features consistent with adenoma/hyperplasia (homogeneous, well circumscribed, lipid-rich with 10 or less Hounsfield units (HU) on unenhanced CT, and for MRI a clear signal intensity decrease on opposed-phase images, compared with in-phase images or if they had other typical benign characteristics such as a thin-walled cyst, myelolipoma or adrenal haemorrhage. Lipid poor (over 10 HU on unenhanced series with an absolute washout with late series measured 15 minutes after contrast injection of more than 50 per cent) small adenomas and large (≥3 cm) lipid rich adenomas were considered benign if they were stationary (<20% or <5 mm increase in diameter) at follow-up of at least 12 months. Lesions that were not evaluated with regard to lipid content (fifty patients that were predominantly included before a change of the local guidelines 2008) were considered benign if they were stationary at 12 months follow-up.

### Statistical methods

To evaluate the validity of an initial normal screening for the identification of individuals with hormone producing tumours, the sensitivity, specificity, positive predictive value and negative predictive value were calculated. The distribution of location (i.e. left or right) of unilateral incidentalomas was presented as proportions (%) associated with 95% confidence intervals (CI) including continuity corrections. The chi-square test was applied to test the differences in proportion of different cardiometabolic risk factors in patients with and without biochemical signs of cortisol hypersecretion. P-values <0.05 were considered significant. Proportions of locations of unilateral incidentalomas were calculated using VassarStats [12]. All other calculations were performed in IBM SPSS Statistics 22.0 (SPSS Inc., Chicago, IL, USA).

## Results

**Figure 1:**
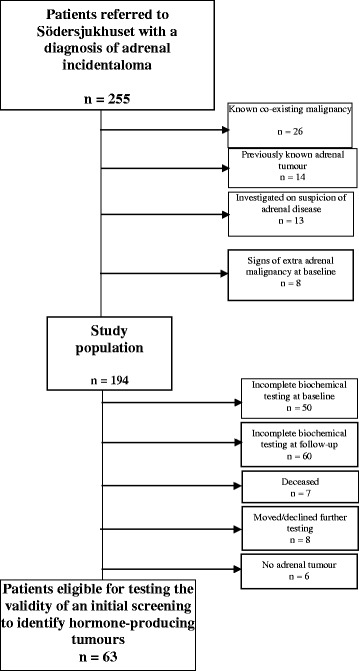
**Patient flow chart.**

Individuals who presented with a first time diagnosis of adrenal incidentaloma were considered eligible for inclusion in the study (n = 255). Patients with known co-existing malignancy (n = 26), with previously known adrenal tumour/s (n = 14), patients who had been investigated on suspicion of adrenal disease (n = 13), or had had signs of extra-adrenal malignancy at baseline (n = 8) were excluded, resulting in a study population of 194 patients (Figure 1).

The patient and adrenal imaging characteristics at the time of the initial visit to the Department of Endocrinology are presented in Table 1. The mean age (SD) was 65 years (12), and 63% were women. Left-sided adrenal tumour was found in 72% of the 156 patients with unilateral incidentaloma. Bilateral incidentalomas were present in 19% of the patients. The mean follow-up time (range) was 24 (0–59) months. Seven individuals (5%) deceased during the follow-up period due to non-adrenal malignancy (n = 2), cardiovascular disease (n = 1), sepsis (n = 1), pneumonia (n = 1), or unknown reason (n = 2).

**Table 1:** **Patient and adrenal characteristics at baseline (n patients = 194, n incidentalomas = 231)**

**Patient characteristics**	
Mean age (SD^a^)	65 (12)
Women, *n*	122 (63%)
Co-morbidity	
*Hypertension, n*	108 (56%)
*Hyperlipidemia, n*	52 (27%)
*Diabetes (type 2), n*	41 (21%)
*COPD* ^*b*^ *, n*	33 (17%)
*Ischemic heart disease, n*	24 (12%)
*Previous history of malignancy, but no evidence of present disease at the time the adrenal incidentaloma was detected*	24 (12%)
*Stroke, n*	11 (6%)
*Heart failure, n*	7 (4%)
*Peripheral arterial diseasecss n*	7 (4%)
**Adrenal characteristics**	
Unilateral incidentaloma (right), *n*	44 (23%)
Unilateral incidentaloma (left), *n*	111 (57%)
Unilateral incidentaloma (side not stated), *n*	1 (0.5%)
Bilateral incidentalomas, *n*	37 (19%)
Mean size at initial adrenal imaging, mm (SD^1^)^c^	21 (9.3)
Lipid rich (≤10 Hounsfield units)^d^	79 (81%)
Lipid poor (>10 Hounsfield units) with an absolute washout < 50% indicating benign tumour^e^	9 (45%)
Lipid poor (>10 Hounsfield units) with an absolute washout ≥ 50% indicating malign tumour^e^	2 (10%)

^a^Standard Deviation.

^b^Chronic Obstructive Lung Disease.

^c^For incidentalomas examined with adrenal imaging where size was stated (*n* = 218).

^d^For incidentalomas were attenuation and washout was quantified (*n* = 98).

^e^For incidentalomas were both attenuation and washout were quantified (*n* = 20).

The outcome of the investigations is presented in Table 2. Of the adrenal incidentalomas with a known diagnosis, 94% were benign, non-hyperfunctioning tumours. Two patients were diagnosed with cortisol hypersecretion (one underwent adrenalectomy while the other have hitherto been conservatively treated), and one with pheochromocytoma. In addition, 6 patients underwent surgery due to large tumour size where subsequent histological examination revealed ganglioneuroma (n = 1), hemorrhagic cyst (n = 1), benign adenoma (n = 3), and accessory spleen (n = 1, i.e. no adrenal tumour). No cases of hyperaldosteronism or adrenocortical cancer were identified.

**Table 2:** **Outcome of the investigations of 231 incidentalomas in the study population (n = 194)**

**Diagnosis**	***n*** ^***a***^	**Comment**	**Outcome related to discovered incidentaloma**
**BENIGN NON-HYPERFUNCTIONING TUMOURS**	160 (94%)		N/A^b^
Adenomas	154 (91%)		N/A
Myelolipoma	3 (1.8%)		N/A
Ganglioneuroma	1 (0.6%)	Diagnosis through PAD^c^.	Surgery.
Hemorrhagic cyst	1 (0.6%)	Diagnosis through PAD.	Surgery.
Hematoma	1 (0.6%)		
**Malignant or functioning tumours**	3 (1.8)		N/A
Cortisol hypersecretion	2 (1.2%)	1 patient underwent surgery due to Cushings syndrome, and PAD showed adrenal hyperplasia.	Surgery (n = 1).
		1 patient s were diagnosed with subclinical Cushing but not judged to benefit from surgery and therefore conservatively treated	Conservative treatment (n = 1)
Pheochromocytoma	1 (0.6%)	Confirmed with PAD	Surgery.
**No adrenal tumour**	6 (3.5%)	Further investigation diagnosed: sarcoma (n = 1), renal cyst (n = 1), ventricular gut diverticulum (n = 1), pleating of the tail of pancreas (n = 1), accessory spleen (n = 1), and no tumour-like structure was found in the adrenal CT (n = 1).	Referred to another department (n = 1, Department of Sarcoma).
			Surgery due to large tumour size, where PAD showed an accessory spleen (n = 1).
**Unknown**	62 (N/A)	Unknown diagnosis due to absence of adequate follow-up (n = 43), death (n = 10) the patient declining further investigation (n = 6), or still ongoing investigations (n = 3)	N/A

^a^Given percentages are based on patients with a known diagnosis (n = 170).

^b^Non Applicable.

^c^Pathological Anatomic Diagnosis.

**Figure 2:**
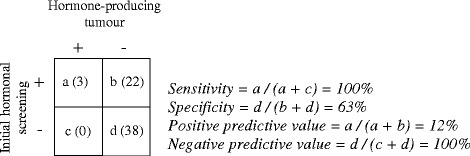
**The validity of an initial hormonal screening to identify individuals with hormonal producing tumours in 63 patients.**

Sixty-three patients underwent a complete biochemical testing both at baseline and at follow up. All three hormone-producing tumours were found among the 25 patients with an abnormal hormonal screening at baseline. Among the 38 patients with an initial normal complete hormonal screening no hormone overproduction was found at 24 months follow-up. Thus, the sensitivity, specificity, positive predictive value and negative predictive values of an initial complete negative screening to predict a hormone producing tumour were 100%, 63%, 12% and 100%, respectively (Figure 2). To further evaluate the biochemical testing we investigated respective hormonal axis separately. For example, when examining the cortisol axis, we included all patients that were investigated adequately with regard to cortisol excretion both at baseline and at 24 months follow-up, even though data on catecholamine or aldosterone secretion was missing. Thus, among 57, 63 and 66 patients with an initial normal cortisol, aldosterone and catecholamine evaluation respectively at baseline, no case of hormonal hyperproduction was discovered at follow-up.

## Discussion

In the present study 94% of the incidentalomas consisted of benign, non-hyperfunctioning adenomas. No adrenocortical cancer or adrenal metastasis was found. None of the patients with an initial negative biochemical screening developed hormonal hypersecretion during the follow-up.

Our results are in accordance with the investigation by Muth *et al.* [13]. In contrast, other studies have indicated a potential of hormonal activation in patients with an initial negative screening and therefore support long-term hormonal monitoring [14-16]. The most common disorder reported during follow-up is the occurrence of autonomous cortisol secretion in patients without clinical signs of Cushing syndrome, i.e. subclinical Cushing syndrome (SCS), while the onset of catecholamine overproduction or hyperaldosteronism after initial negative screening is very rare [2]. Given that the investigation of cortisol secretion is associated with methodological problems causing a high proportion of false positive results [17], that cortisol levels may vary over time [18], and the questionable benefit of performing adrenalectomy in patients with SCS [19], conservative treatment may be a better strategy.

The high proportion of benign, non-hyperfunctioning adenomas of 94% found in the present study was in accordance with some previous studies but not all. Two Swedish prospective studies estimated corresponding prevalences of 96% (218/226) and 91% (347/381) respectively [13,20], while most other studies indicate a prevalence around approximately 80% [2]. The explanation of these discrepancies is probably that some studies may be biased towards an unhealthier group of patients treated in specialised care units with a higher prevalence of hormonal aberrations.

The age of the individuals involved in the current study range from 34 to 94 with few individuals below 50 (n = 16) years and the results of the study may therefore not be extrapolated to a younger population. The prevalence of adrenal incidentalomas is rare in individuals < 40 years old, and the chance of finding a hormonal disturbance or congenital condition such as congenital adrenal hyperplasia may be increased [21] but this has to be studied further.

An interesting finding was the discovery that left-sided adrenal incidentalomas were more than twice as common as right-sided ones. This is in contrast to previous studies [22,23], and it could be speculated that the liver impairs the visualising of the right adrenal gland.

The retrospective design investigating the work-up of incidentalomas diagnosed during a five year period may have hampered the study to some extent. Due to a shift towards the use of the overnight low-dose (1 mg) DST and plasma metanephrines rather than 24-hour urinary collections of cortisol and catecholamines, the patients were not uniformly investigated which may question the validity of the data. For various reasons, the number of patients eligible to study the validity of an initial normal hormonal screening to identify hormone-producing tumours was reduced to 63 which weakens the conclusions (Figure 1). On the other hand, a strength with the present study is that the included patients were referred from a defined catchment area where Södersjukhuset represents the only Endocrine out-patient clinic investigating patients with adrenal incidentalomas. The study population may therefore represent the population in general very well. This is in contrast to some previous studies performed at tertiary centers which increases the risk for selection bias towards a less healthy study population, and findings of rare syndromes [2,21]. In order to further evaluate the value of long term biochemical follow up we do suggest prospective larger studies.

## Conclusion

The vast majority of the adrenal incidentalomas were benign, hormonally inactive adenomas. For patients with an initial normal biochemical investigation, the 24-month hormonal follow-up did not result in the finding of any additional hormonally active tumours. This study indicates that patients with an initial normal hormonal screening may not need further biochemical follow-up.
